# Recent atmospheric changes and future projections along the Saudi Arabian Red Sea Coast

**DOI:** 10.1038/s41598-021-04200-z

**Published:** 2022-01-07

**Authors:** Abdulhakim Bawadekji, Kareem Tonbol, Nejib Ghazouani, Nidhal Becheikh, Mohamed Shaltout

**Affiliations:** 1 College of Science, Northern Border University, Arar, Saudi Arabia; 2 College of Maritime Transport and Technology, Arab Academy for Science, Technology and Maritime Transport, Abu-Qir, Alexandria Egypt; 3 College of Engineering, Northern Border University, Arar, Saudi Arabia; 4 Oceanography Department, Faculty of Science,, Alexandria University, Alexandria, Egypt

**Keywords:** Atmospheric science, Climate change, Climate sciences

## Abstract

Recent and future climate diagrams (surface air temperature, surface relative humidity, surface wind, and mean sea level pressure) for the Saudi Arabian Red Sea Coast are analysed based on hourly observations (2016–2020) and hourly ERA5 data (1979–2020) with daily GFDL mini-ensemble means (2006–2100). Moreover, GFDL mini-ensemble means are calculated based on the results of three GFDL simulations (GFDL-CM3, GFDL-ESM2M, and GFDL-ESM2G). Observation data are employed to describe the short-term current weather variability. However, ERA5 data are considered to study the long-term current weather variability after bias removal via a comparison to observations. Finally, a bias correction statistical model was developed by matching the cumulative distribution functions (CDFs) of corrected ERA5 and mini-ensemble mean data over 15 years (2006–2020). The obtained local statistic were used to statically downscale GFDL mini-ensemble means to study the future uncertainty in the atmospheric parameters studied. There occurred significant spatial variability across the study area, especially regarding the surface air temperature and relative humidity, based on monthly analysis of both observation and ERA5 data. Moreover, the results indicated that the ERA5 data suitably describe Tabuk, Jeddah and Jizan weather conditions with a marked spatial variability. The best performance of ERA5 surface air temperature and relative humidity (surface wind speed and sea level pressure) data was detected in Tabuk (Jeddah). These data for the Saudi Arabian Red Sea coast, 1979–2020, exhibit significant positive trends of the surface air temperature and surface wind speed and significant negative trends of the relative humidity and sea level pressure. The GFDL mini-ensemble mean projection result, up to 2100, contains a significant bias in the studied weather parameters. This is partly attributed to the coarse GFDL resolution (2° × 2°). After bias removal, the statistically downscaled simulations based on the GFDL mini-ensemble mean indicate that the climate in the study area will experience significant changes with a large range of uncertainty according to the considered scenario and regional variations.

## Introduction

**Figure 1:**
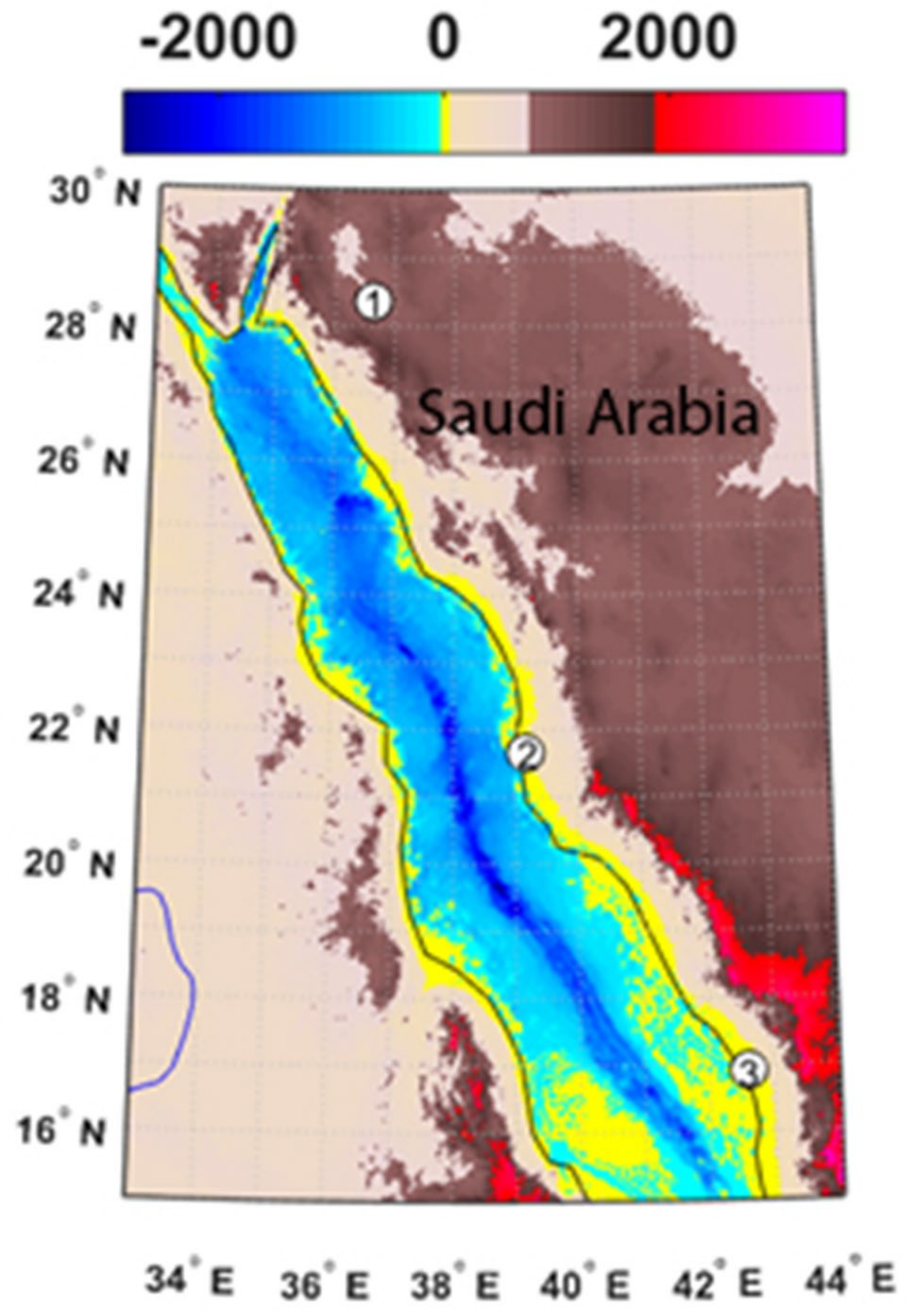
Digital elevation chart of the study area with the location of Tabuk (1), Jeddah (2) and Jizan (3) weather observation stations. Math Works, Inc. MATLAB. Version 2020a, Math Works, Inc., 2020. www.mathworks.com.

The Saudi Arabian Red Sea Coast extends approximately 1800 km from north of Tabuk (28.75° N, 34.83° E) in the north to south of Jizan (16.15° N, 42.75° E) in the south, as shown in Fig. 1. This coastal zone includes four large cities, namely, Tabuk, Al Madinah, Jeddah and Jizan, and religious and historical sites, tourist resorts, fertile agricultural lands, and economic resources, such as oil and gas resources, in addition to fishing activities. The current study focuses on three sites along the Saudi Arabian Red Sea Coast: Tabuk in the north, Jeddah at the centre and Jizan in the south.

Tabuk (28.365° N, 36.619° E), which is located in the northwestern region of Saudi Arabia at an elevation of 760 m above mean sea level (Fig. 1), is classified as an agricultural area depending mostly on groundwater. However, Jeddah (21.67° N, 39.15° E), which is the second largest city in the kingdom (Fig. 1), suffers from a high population density and rapid urbanization. Thus, Jedda is considered vulnerable to climate change, and frequent flooding is a current example^1^. Moreover, Jizan (16.9° N, 42.6° E), which is located in the southwest corner of Saudi Arabia, is considered the richest agricultural region in Saudi Arabia. In addition, Jizan is projected to experience damage resulting from projected warming trends in the future^2^.

According to^3,4^, Tabuk is considered a hyper-arid (extremely arid) zone, where the aridity index (annual precipitation/potential evapotranspiration) is lower than 0.03. However, Jeddah and Jizan are considered arid zones with aridity index values between 0.03 and 0.2. These values correspond to a notable diurnal temperature cycle. In addition, according to the Köppen–Geiger climate classification, the Tabuk, Jeddah and Jizan climates are classified as BWh climates (the main climate is arid, the precipitation type is the desert type, and the temperature is high).

According to^6^, the annual average air temperatures (1978–2010) over Tabuk, Jeddah and Jizan reach 22.15 °C, 27.84 °C, and 30.64 °C, respectively. Almazroui et al.^7^ studied the mean annual temperature/rainfall throughout Saudi Arabia based on 27 stationary (including Tabuk, Jeddah and Jizan) data observations from 1979 to 2009. They reported that the annual mean surface air temperature (T2m) along the Saudi Arabian Red Sea Coast ranges from 24 to 30 °C. Furthermore, Almazroui^8^ simulated weather characteristics from 2001 to 2005 with the RegCM model over the MENA CORDEX domain, including the study area. They determined that the simulated daily average surface air temperature ranges from 18 to 21 °C in Tabuk and ranges from 21 to 30 °C in Jeddah and Jizan. More recently, Azamathulla et al.^9^ employed four climate parameters (atmospheric pressure, wind speed, relative humidity, and rainfall) to efficiently project the future air temperature in Tabuk by developing two different generic models: artificial neural network and gene expression programming models. Alrashed and Asif^10^ found that the annual climatic average air temperature over Jeddah reaches 27.9 °C.

According to^11^, the Arabian Peninsula experienced a warm trend of 0.055 °C decade^−1^ during the period from 1879 to 1992. Moreover, Almazroui et al.^7^ observed that Saudi Arabia experienced a warming trend of 0.06 °C decade^−1^ during the 1978–2009 period.

Azamathulla et al.^9^ reported that the annual average wind speed (relative humidity) over Tabuk reached 5.5 m s^−1^ (32.9%) based on monthly average climate data from 1986 to 2015. However, Alrashed and Asif^10^ determined that the annual climatic average wind speed (relative humidity) over Jeddah reached 2.3 m s^−1^ (65%). In addition, Nassar et al.^12^ stated that the relative humidity over Jizan ranged from 54% in July to 68.5% in September.

Many researchers have indicated that the air temperature, wind field, relative humidity and atmospheric pressure are the most important climate change measurements considering their direct effect on all life sectors. The air temperature is considered one of the most important climate factors, as the daily activities of people are influenced by even a minor change in air temperature^13^. According to Maia-Silva et al.^14^, relative humidity (RH; calculated from the surface air and dew point temperatures) rise is one of the main climate challenges, as the increase in surface air temperature is correlated with a decrease in relative humidity. This coupling effect can be disastrous, especially during heat wave events. This may increase mortality/morbidity rates. However, wind fields and the progress associated with wind technology may reduce CO_2_ emissions^15^. In general, changes in mean sea level pressure (SLP) could impose a notable effect on climate because SLP controls atmospheric circulation. Therefore, this factor influences wind, moisture movement, precipitation, and temperature variabilities.

In 2000, IPCC^16^ introduced the Special Report on Emissions Scenarios (SRES), covering a range of greenhouse gas (GHG) emissions (A1B, A2, and B1 scenarios) under the Coupled Model Intercomparison Project, phase three (CMIP3). Meehl et al.^17^ reported that based on the success of SRES scenarios, the IPCC elevated the CMIP3 to the CMIP5 in late 2008 and described new future scenarios (RCP2.6, RCP4.5, RCP6.0, and RCP8.5). These new future RCP scenarios comprise various RCP combinations. RCP denotes the representative concentration pathway, and the numbers indicate the assumed radiative forcing by the end of the twenty-first century.

The current research examines the current and future climate conditions along the Saudi Arabian Red Sea Coast. First, observation data were employed to describe the current short-term weather variability from 2006 to 2020. Second, the current research qualifies the use of ERA5 data in capturing the surface air temperature (T2m), relative humidity (RH), surface wind field and mean sea level pressure. Third, 42 years (1979–2020) of ERA5 data after bias removal were analysed to describe the current long-term climate. Finally, the future climate in the study area was projected via statistical analyses through the above four CMIP5 scenarios. The materials and methods adopted are presented in “Results” section, the results and discussion are provided in “Discussion and Conclusions” section, and the conclusions are outlined in “Future work” section.

## Results

### Observed data

#### Monthly time series

**Figure 2:**
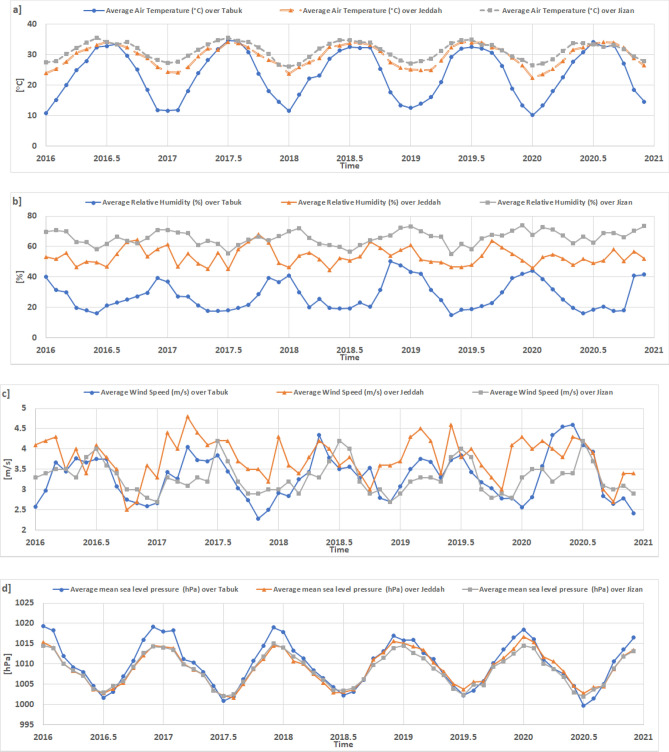
Monthly average time series for the parameters studied based on hourly observation data (2016–2020) in Tabuk, Jeddah and Jizan. Microsoft Office (Excel). Version 365. https://www.office.com/.

Monthly average time series for T2m, RH, WS_10_, and SLP based on hourly observation data (2016–2020) for Tabuk, Jeddah and Jizan are shown in Fig. 2.

In Tabuk, the T2m data revealed that January 2020 (10.16 °C) is the coldest month, while July 2017 is the warmest month (34.68 °C), as shown in Fig. 2a. Similarly, the RH data revealed that November 2018 exhibits the maximum monthly RH mean value (50.25%), while May 2019 attains the lowest monthly RH mean value (14.9%), as shown in Fig. 2b. In addition, the WS_10_ data revealed that June 2020 is the windiest month (4.6 m s^–1^), while November 2017 is the calmest month (2.29 m s^–1^), as shown in Fig. 2c. Generally, the SLP data revealed that January 2016 attains the maximum monthly SLP value (1019.33 hPa), while July 2020 exhibits the lowest SLP value (999.74 hPa), as shown in Fig. 2d.

In Jeddah, the T2m data indicate that January 2020 (22.3 °C) is the coldest month, while August 2020 (34.2 °C) is the warmest month. Similarly, the RH data confirm that October 2017 (68.0%) attains the maximum monthly RH mean value, while May 2018 (44.5%) exhibits the lowest mean value. Moreover, the WS_10_ data reveal that April 2017 (4.8 m s^–1^) is the windiest month, while October 2016 (2.5 m s^–1^) is the calmest month. Generally, the SLP data reveal that January 2020 (1016.7) attains the maximum monthly SLP value, while August 2017 (1001.7 hPa) exhibits the lowest monthly SLP value.

In Jizan, the T2m data confirmed that the maximum (monthly) average value was 35.05 °C (26.1 °C). The maximum (minimum) value occurred in June 2020 (January 2019). Similarly, the RH data confirmed that December 2019 (74.1%) attained the maximum monthly RH mean value, while May 2019 (55%) attained the lowest monthly RH mean value. Moreover, the WS_10_ data revealed that the highest (lowest) average wind speed value is 4.2 m s^–1^ (2.7 m s^–1^). The windiest (calmest) month is July 2020 (January 2020). Generally, the SLP data reveal that December 2017 (1015 hPa) attains the maximum monthly SLP value, while July 2020 (1002.0 hPa) achieves the lowest monthly SLP value.

Generally, T2m increased from Jizan to Tabuk. The highest temperatures were close among the three studied cities, while the lowest temperature markedly occurred in Tabuk (11 °C) rather than in Jeddah (24 °C) and Jizan (27 °C). In contrast, RH increased from Jizan to Tabuk, and there was a significant difference between the maximum and minimum RH values among the three studied cities. There was no significant difference among Tabuk, Jeddah and Jizan regarding the WS_10_ regime. Regarding SLP, Jizan and Jeddah exhibited similar behaviour. However, SLP in Tabuk indicated markedly higher values than those in Jizan/Tabuk.

#### Annual, monthly, and hourly cycles

In Jeddah and Jizan, the hottest (coldest) year was 2016 (2020). However, in Tabuk, the hottest (coldest) year was 2018 (2019). The highest/lowest annual average RH values in the study area changed from city to city, similar to WS_10_. However, the SLP annual pattern in the study area revealed similarities between Tabuk and Jizan, where the maximum (minimum) annual SLP value occurred in 2016 (2020). In Jeddah, the maximum (minimum) annual value occurred in 2019 (2017), as indicated in Table 1.

**Table 1:** Annual, monthly, and hourly characteristics of the parameters studied based on the hourly observation data (2016–2020) in Tabuk, Jeddah and Jizan.

Variables		Tabuk		Jeddah		Jizan	
		Maximum	Minimum	Maximum	Minimum	Maximum	Minimum
Surface air temperature (T2m, °C)	Annual	2018 (23.99 °C)	2019 (23.25 °C)	2016 (29.8 °C)	2020 (29.4 °C)	2016 (31.6 °C)	2020 (30.8 °C)
	Monthly	July 33.3 °C	January 11.3 °C	July 33.95 °C	January 23.87 °C	June 34.7 °C	January 26.9 °C
	Hourly	30.4 °C at 15:00	16.86 °C at 6:00	33.38 °C at 12:00	25.97 °C at 5:00	34.19 °C at 12:00	28.36 °C at 5:00
Relative humidity (RH, %)	Annual	2019 (29.06%)	2017 (26.1%)	2017 55%	2020 51.9%	2020 68%	2017 64.5%
	Monthly	December 41.46%	June 17.53%	September 62.30%	May 46.84%	December 71.30%	July 58.9%
	Hourly	42.76% at 6:00	16.01% at 15:00	65.84% at 5:00	40.55% at 11:00	74.0% at 5:00	57.0% at 12:00
Surface wind speed (WS_10_, m s^–1^)	Annual	2020 (3.43 m s^–1^)	2016/2017 (3.22 m s^–1^)	2017/2019 (3.9 m s^–1^)	2016 (3.6 m s^–1^)	2016/2020 (3.4 m s^–1^)	2017 (3.2 m s^–1^)
	Monthly	May 3.95 m s^–1^	December 2.60 m s^–1^	February 4.1 m s^–1^	October 3.0 m s^–1^	July 4.1 m s^–1^	December 2.9 m s^–1^
	Hourly	4.69 m s^–1^ at 17:00	2.26 m s^–1^ at 6:00	6.13 m s^–1^ at 14:00	2.1 m s^–1^ at 5:00	4.69 m s–1 at 17:00	2.26 m s^–1^ at 6:00
Mean sea level pressure (SLP, hPa)	Annual	2016 (1010.7 hPa)	2020 (1009.4 hPa)	2019 1009.7 hPa	2017 1008.4 hPa	2016 1009.0 hPa	2020 1008.4 hPa
	Monthly	January 1017.9 hPa	July 1001.41 hPa	January 1015.1 hPa	July 1002.9 hPa	January 1014.3 hPa	July 1002.6 hPa
	Hourly	1011.9 hPa at 8:00	1001.8 hPa at 15:00	1010.3 hPa at 8:00	1007.8 hPa at 17:00	1010.3 hPa at 11:00	1006.2 hPa at 19:00

**Figure 3:**
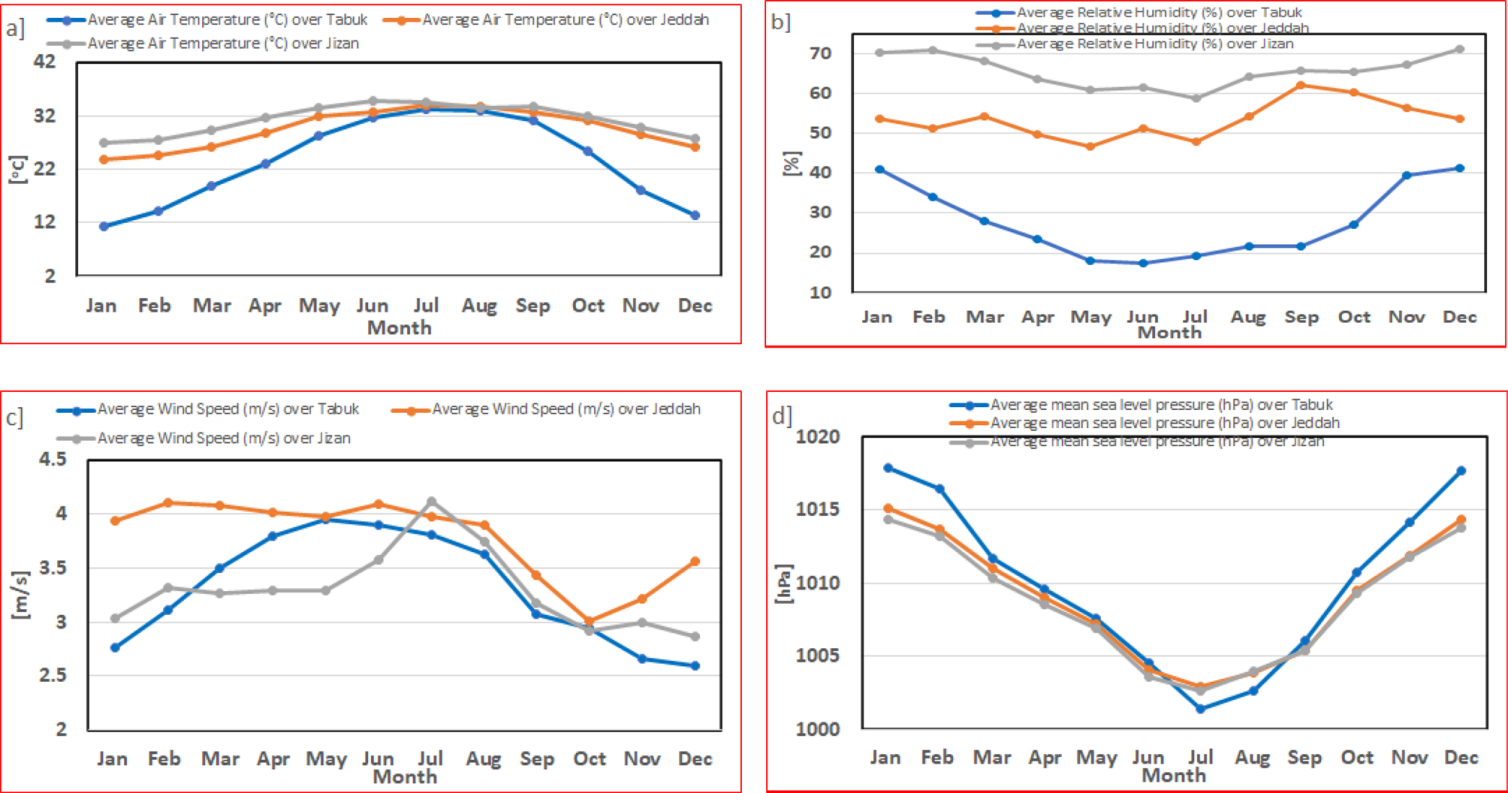
Observed short-term monthly means (annual cycle) for the parameters studied based on the hourly observed data (2016–2020).

In terms of the annual cycle (climate monthly average), as described in Table 1 and Fig. 3, the maximum T2m value occurred in July for Tabuk and Jeddah and occurred in June over Jizan. In contrast, the minimum T2m value occurred in January in the three studied cities. There was no common pattern among the three studied cites regarding RH and WS_10_. In contrast, the SLP annual cycles were similar among Tabuk, Jeddah and Jizan, as the maximum (minimum) values occurred in January (July).

**Figure 4:**
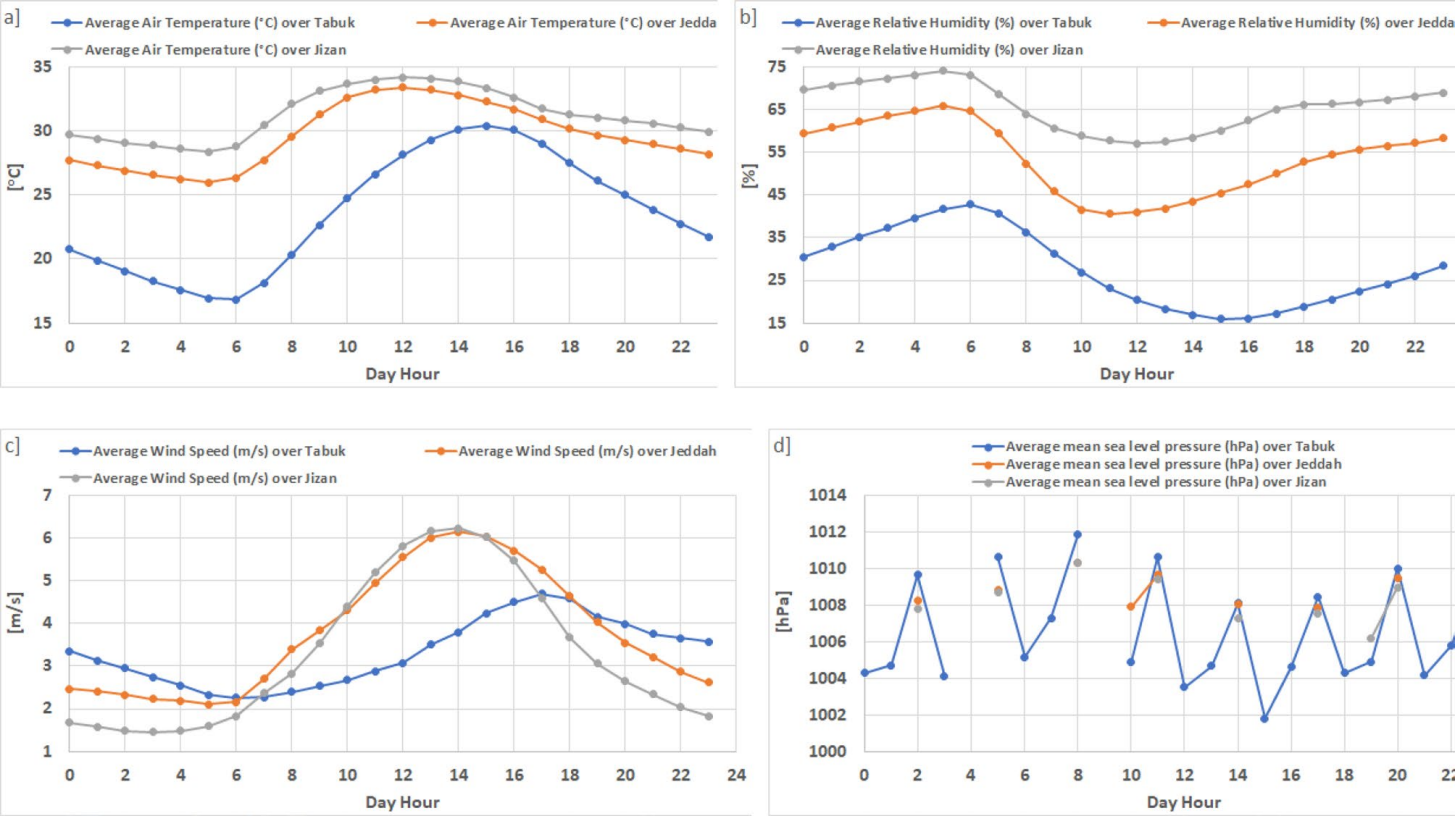
Observed short-term hourly means (daily cycle) for the parameters studied based on the hourly observed data (2016–2020) (there is a large gap in the mean sea level observation data for Jizan and Jeddah). Microsoft Office (Excel). Version 365. https://www.office.com/.

The daily T2m cycle (Table 1; Fig. 4) amplitude in Tabuk (13.5 °C) is much higher than that in Jeddah (7.1 °C) and Jizan (5.3 °C). Similarly, the amplitude of the RH daily cycle in Tabuk (26.7%) is much higher than that in Jeddah (25.2%) and Jizan (17%). The amplitude of the WS_10_ daily cycle reaches its maximum value (4.7 m s^−1^) in Jizan followed by (4 m s^−1^) Jeddah and (2.4 m s^−1^) Tabuk. Finally, the observed short-term SLP hourly means indicate a significant hourly variation ranging from 1011.88 hPa at 8:00 to 1001.8 hPa at 15:00, as shown in Tabuk. The daily SLP cycle cannot be analysed in Jizan and Jeddah based on the available observed data due to a large number of missing observations.

In Tabuk, the annual WS_10_ cycle reveals a direct correlation (n = 12, R = 0.69) with T2m and inverse correlations (n = 12, R = − 0.88) with RH and (n = 12, R = − 0.76) SLP. Similar to Tabuk, the annual WS_10_ cycle in Jizan indicates a significant direct correlation with T2m and significant inverse correlations with RH and SLP. In contrast, the annual WS_10_ cycle in Jeddah reveals a non-significant correlation with T2m and SLP, while WS_10_ achieves a significant inverse correlation with RH (n = 12, R = − 0.76). This indicates that the four parameters studied are co-dependent only in Tabuk and Jizan. In addition, in Tabuk, RH monthly maximum values occurred in December. After one month (in January), SLP reached its monthly maximum values. After 4 months (in May), WS_10_ reached its monthly maximum values. After 2 months (in July), T2m attained its monthly maximum values, as shown in Fig. 3. Similarly, in Jizan, RH monthly maximum values occurred in December. After one month (in January), SLP reached its monthly maximum values. After 6 months (in June), T2m attained its monthly maximum values. After one month (during July), WS_10_ reached its monthly maximum values.

In contrast, regarding the daily cycle in Tabuk, WS_10_ attains a direct correlation (n = 24, R = 0.76) with T2m and an inverse correlation (n = 24, R = − 0.85) with RH. Moreover, T2m attains an inverse correlation (n = 24, R = − 0.97) with RH. Moreover, SLP exhibits a weak correlation with WS_10_, T2m, and RH, indicating that the daily cycle, which distinguishes WS_10_, T2m, and RH, is not significant for SLP (SLP is distinguished by a 3-h cycle), as shown in Fig. 4. In Jizan and Jeddah, the daily RH, T2m and WS_10_ cycles indicate a significant correlation (n = 24, R > 0.9 between any two parameters. This confirms the previous finding whereby T2m, RH and WS_10_ are co-dependent in the study area. Due to the high percentage of missing SLP data, the hourly correlation between SLP and the studied parameters could not be determined.

#### 10-m height wind direction (WD10)

**Figure 5:**
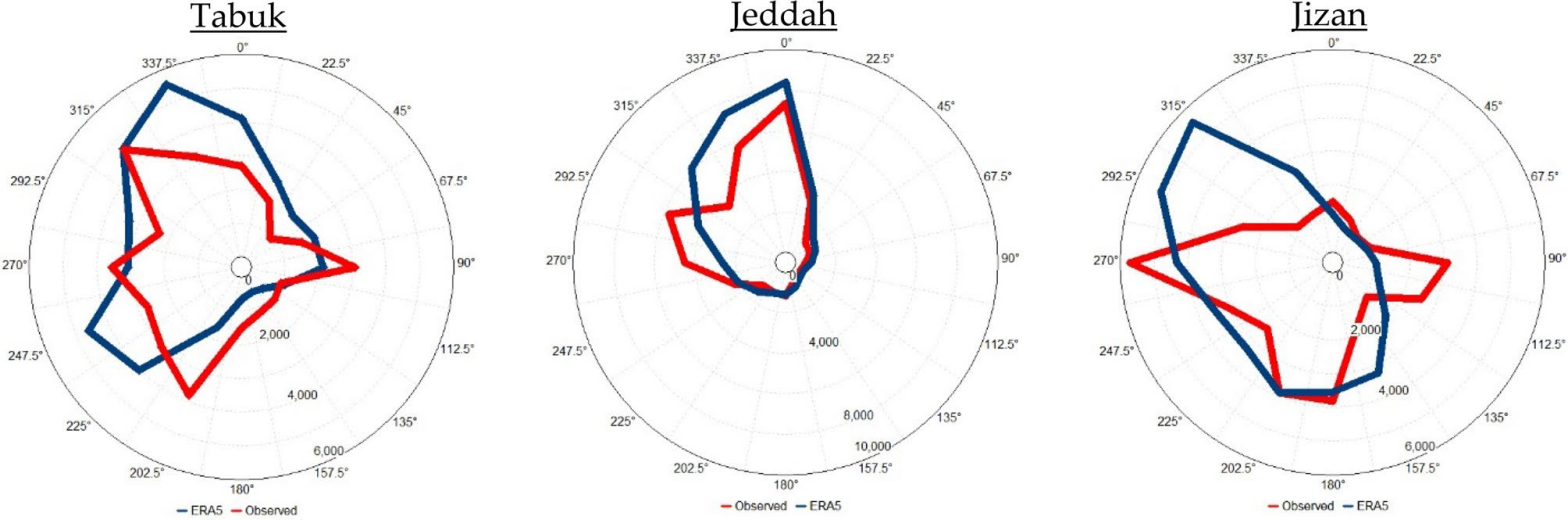
Observed (ERA5) annual wind rose diagram based on hourly observed (ERA5) data from 2016 to 2020 in Tabuk, Jeddah and Jizan. The blue colour indicates the observations, while the red colour indicates the ERA5 data. UL, Inc. Windographer professional edition version 5.0.5. https://www.ul.com/resources/apps/windographer.

The prevailing annual wind direction originated from the northwest (12.2% of the time) followed by the south-southwest direction (10.2% of the time) in Tabuk, as shown in Fig. 5. However, the prevailing annual wind direction originated from the north (19.1% of the time), followed by both the west-northwest and north-northwest directions (14.7% of the time each). In Jizan, the wind rose diagram shows a different pattern than that in Jeddah and Tabuk, where the prevailing annual wind direction originated from the west (15.6% of the time) followed by both the south and south-southwest directions (10.3% of the time each).

**Figure 6:**
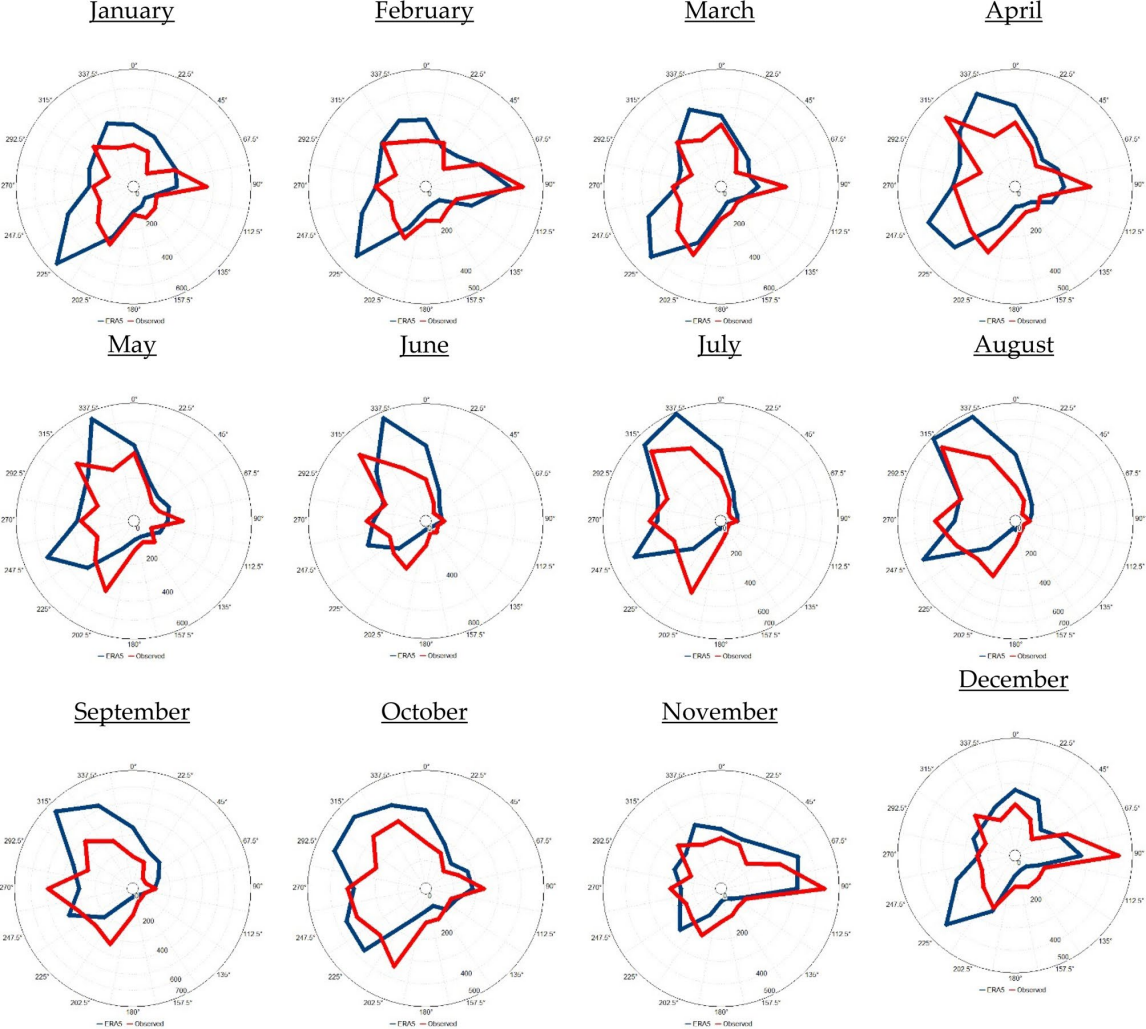
Observed (ERA5) monthly wind rose diagram based on hourly observed (ERA5) data from 2016 to 2020 in Tabuk. The blue colour indicates the observations, while the red colour indicates the ERA5 data. UL, Inc. Windographer professional edition version 5.0.5. https://www.ul.com/resources/apps/windographer.

**Figure 7:**
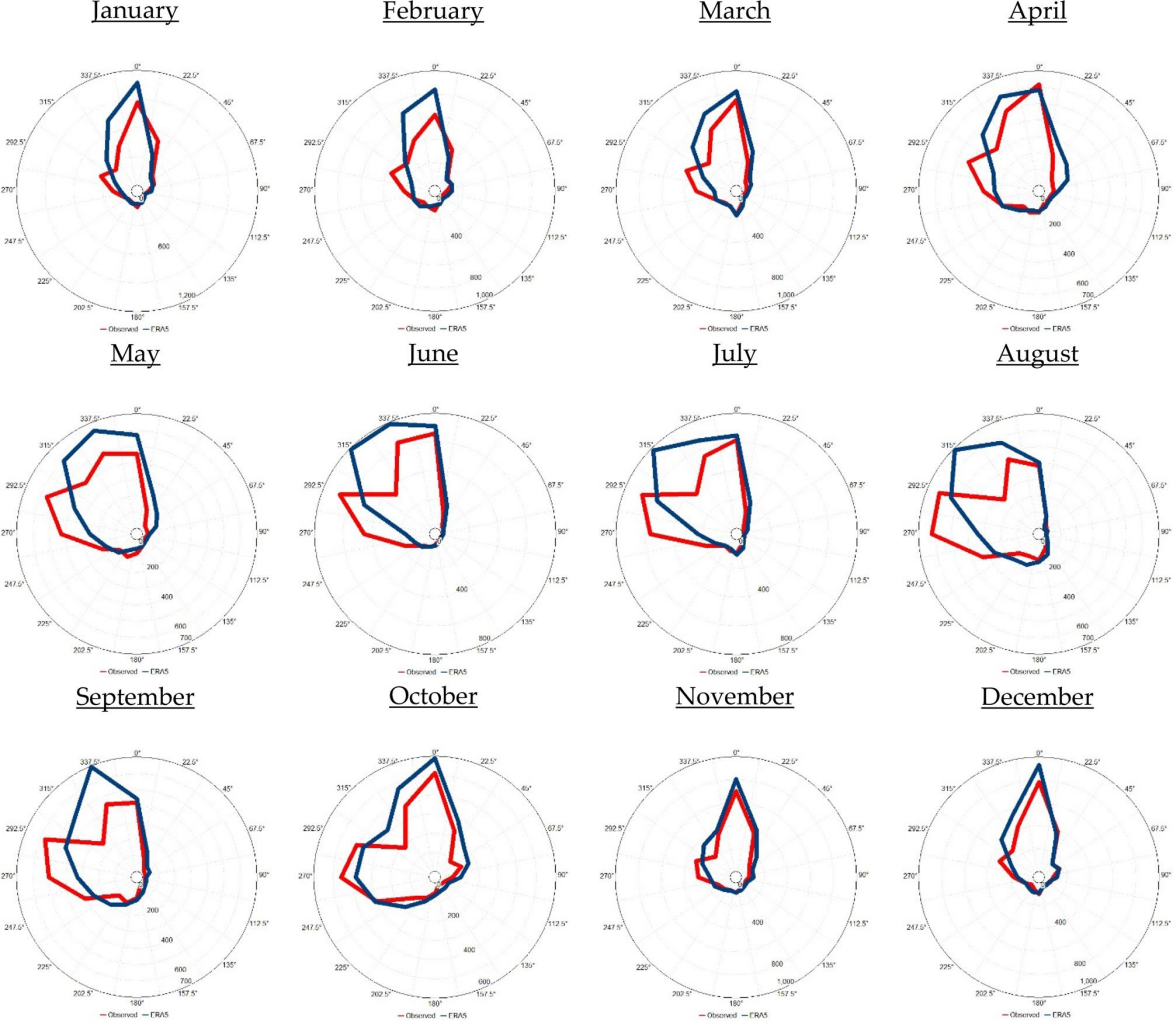
Observed (ERA5) monthly wind rose diagram based on the hourly observed (ERA5) data from 2016 to 2020 in Jeddah. The blue colour indicates the observations, while the red colour indicates the ERA5 data. UL, Inc. Windographer professional edition version 5.0.5. https://www.ul.com/resources/apps/windographer.

In Tabuk, as shown in Fig. 6, in November, December, January, and February, the prevailing monthly wind direction originated from the east for 15%, 16%, 12%, and 14%, respectively, of the time. In March, the prevailing monthly wind direction originated from the south-southwest direction (11% of the time). In April, June, July, and August, the prevailing monthly wind direction originated from the northwest direction at frequencies of 13%, 19%, 16%, and 19%, respectively, of the time. In May, the prevailing monthly wind direction originated from the northwest and south-southwest directions (11% of the time each) at equal frequencies. In August, the prevailing monthly wind direction originated from the west and south-southwest directions at equal frequencies (11% of the time each). In Jeddah (Fig. 7), from October until April, the prevailing monthly wind direction originated from the north. In the months from May to August, the prevailing monthly wind direction originated from the west-northwest.

**Figure 8:**
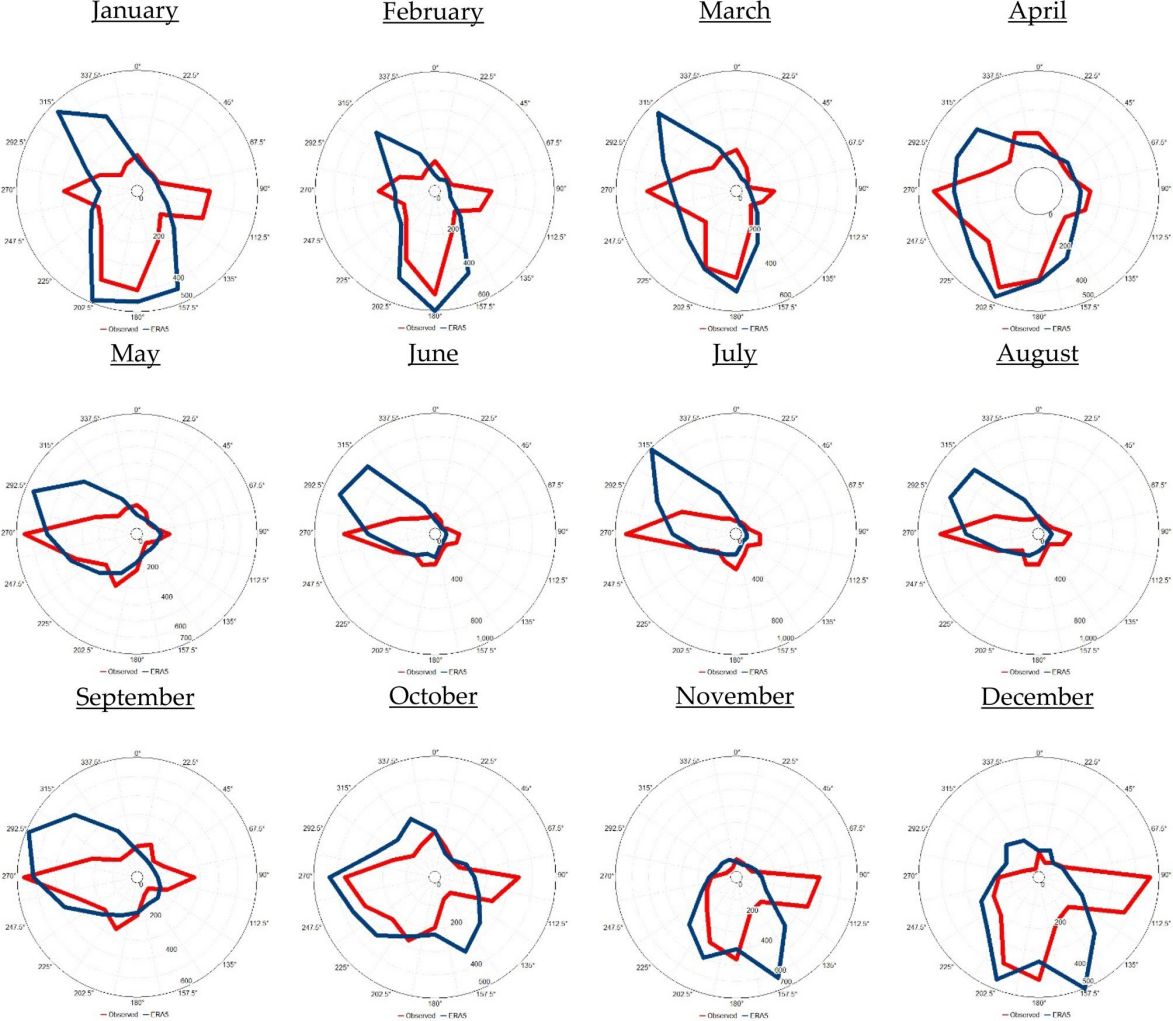
Observed (ERA5) monthly wind rose diagram based on the hourly observed (ERA5) data from 2016 to 2020 in Jizan. The blue colour indicates the observations, while the red colour indicates the ERA5 data. UL, Inc. Windographer professional edition version 5.0.5. https://www.ul.com/resources/apps/windographer.

In Jizan (Fig. 8), in January, February and March, the prevailing monthly wind direction originated from the south. In the months from April to September, the prevailing monthly wind direction originated from the west. From October to December, the prevailing monthly wind direction originated from the east.

### ERA5

#### ERA5 verification

To evaluate the feasibility of using ERA5 data in describing the selected atmospheric parameters in Tabuk, Jeddah and Jizan, a comparison was carried out between ERA5 data and observations covering the observation period from 2016 to 2020 (Table 2; Figs. 6, 7, 8).

**Table 2:** Comparison analysis between the observed and ERA5-based weather variables in Tabuk, Jeddah and Jizan: n = number of observations, and R = correlation coefficient.

Variables			n	R [%]	Minimum	Maximum	Annual mean ± standard deviation
Surface air temperature (T2m, °C)	Tabuk	Observed	42,139	99.3	− 2.0	46.0	23.7 ± 9.5
		ERA5			− 2.3	43.7	22.6 ± 9.3
	Jeddah	Observed	42,699	96.6	15	49	29.6 ± 4.7
		ERA5			15.8	43.8	29.0 ± 3.9
	Jizan	Observed	42,748	80.7	17	42	31.2 ± 3.6
		ERA5			22.4	34.3	29.3 ± 2.3
Relative humidity (RH, %)	Tabuk	Observed	42,127	92.6	4.6	187.6	27.4 ± 16.0
		ERA5			3.9	95.6	33.12 ± 17.1
	Jeddah	Observed	42,625	76.1	4	100	55.5 ± 15.3
		ERA5			17.8	104	69.4 ± 11.1
	Jizan	Observed	42,741	39.5	22.6	100	65.7 ± 10.1
		ERA5			41.4	92.8	76.1 ± 5.2
Surface wind speed (WS_10_, m s^−1^)	Tabuk	Observed	42,128	66.8	0.0	28.8	3.3 ± 2.3
		ERA5			0.0	14.9	2.8 ± 1.6
	Jeddah	Observed	42,730	73.0	0	27.8	3.37 ± 2.3
		ERA5			0	12.9	4.5 ± 2.1
	Jizan	Observed	42,752	58.6	0	23.2	3.3 ± 2.3
		ERA5			0	13.5	3.6 ± 2.0
Mean sea level pressure (SLP, hPa)	Tabuk	Observed	8363	98.4	994.4	1031.0	1009.8 ± 6.5
		ERA5			997.1	1033.1	1012.5 ± 5.6
	Jeddah	Observed	**8476**	99.8	997.1	1022.6	1008.9 ± 4.6
		ERA5			999.8	1022.6	1009.1 ± 4.55
	Jizan	Observed	**8607**	97.5	997.9	1020.3	1008.6 ± 4.4
		ERA5			997.6	1021.1	1008.7 ± 4.3

Generally, the ERA5 data closely matched the observations in the studied cities. With ERA5 data, T2m and WS_10_ were underestimated, while RH and SLP were overestimated, as indicated in Table 2. Moreover, the applied statistical tests (t and f tests) indicated that the observed and ERA5 T2m, RH, WS_10_, and SLP values originated from two equal distributions based on the mean and variance at a 99% significance level. In addition, based on Figs. 6, 7 and 8, it is clear that the observed wind direction agrees with the ERA5-based wind direction. In general, with the ERA5 data, the simulation efficiency in Jeddah was higher than that in Tabuk, with the lowest simulation efficiency in Jizan.

#### ERA5 bias correction

**Figure 9:**
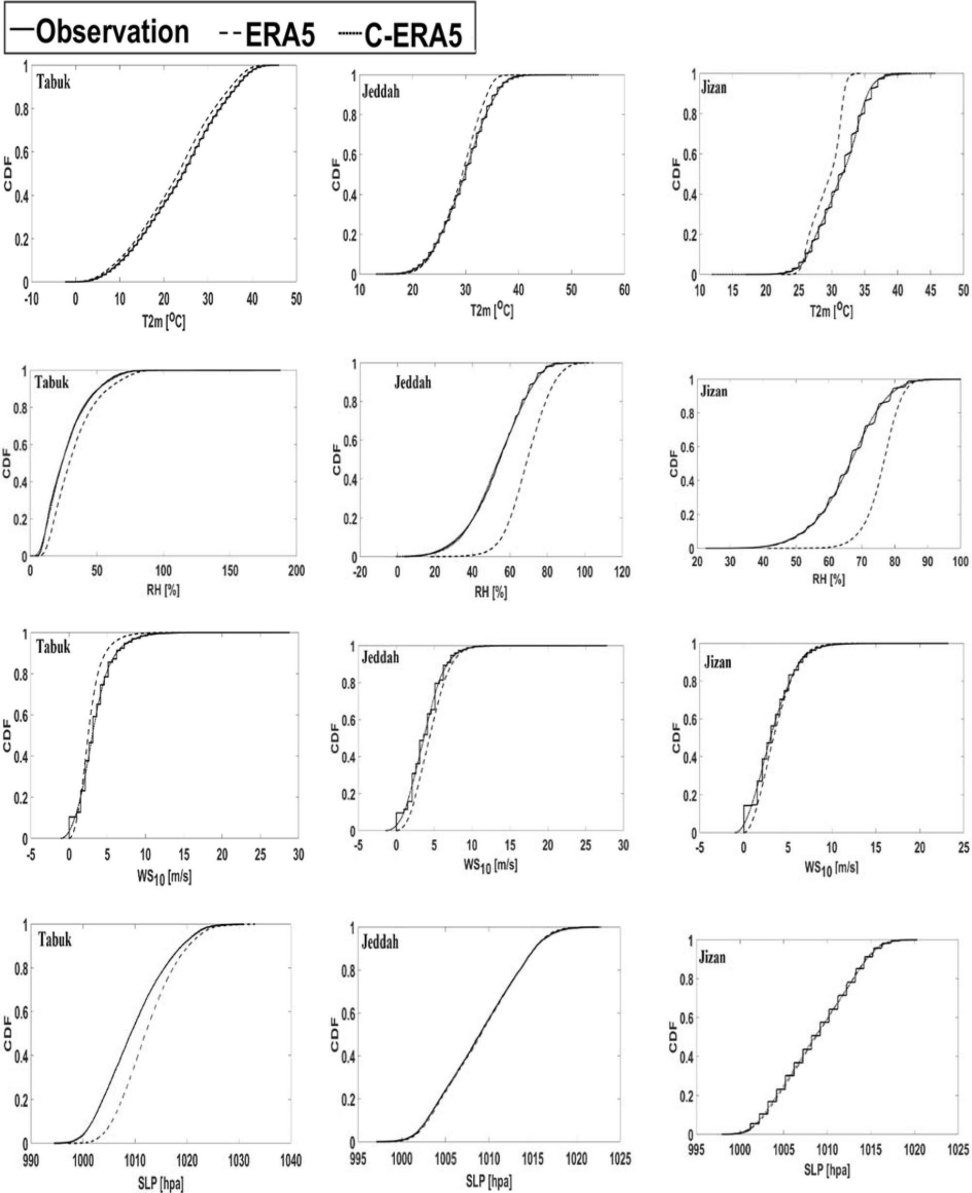
Cumulative distribution function (CDF) between the observations and ERA5 data from 2016–2020 for T2m (2-m air temperature), RH (relative humidity), surface wind speed (WS_10_), and SLP (mean sea level pressure). The corrected ERA5 (C_ERA5) curves are identical to the observed curves. Math Works, Inc. MATLAB. Version 2020a, Math Works, Inc., 2020. www.mathworks.com.

To remove the bias in the ERA5 data, CDF bias correction was applied to match the CDF of the observations to that of the ERA5 data from 2016 to 2020 (Fig. 9). This strategy conserves the nature of the data while maintaining the same correlation values and adjusts the bias to zero. This strategy was applied to the long-term ERA5 data to obtain corrected ERA5 data (C_ERA5) for Tabuk, Jeddah and Jizan. Figure 9 confirms the current findings that the ERA5 data more reasonably simulate the current atmospheric parameters in Jeddah and Tabuk than is achieved in Jizan.

#### C_ERA5 statistical analyses

##### Annual mean and trend analyses

The C_ERA5 data (1979–2020) revealed that the annual average T2m, RH and SLP values across the study area exhibits significant spatial variation (Table 3), where T2m and RH increased meridionally from north to south, while SLP decreased meridionally from north to south. The annual long-term WS_10_ average value was very close between Tabuk and Jizan. However, Jeddah was much windier.

**Table 3:** Long-term annual mean and trend analyses of the corrected ERA5 weather variables in Tabuk, Jeddah and Jizan from 1979 to 2020.

Variables		Annual mean ± standard deviation	Trend	Significant (monotonic) trend
Surface air temperature (T2m, °C)	Tabuk	22.4 ± 9.3 °C	0.49 °C decade^−1^	Yes
	Jeddah	28.4 ± 4.7 °C	0.42 °C decade^−1^	Yes
	Jizan	30.7 ± 3.7 °C	0.33 °C decade^−1^	Yes
Relative humidity (RH, %)	Tabuk	29.1 ± 16.7%	− 0.34% decade^−1^	Yes
	Jeddah	54.5 ± 15.5%	− 0.33% decade^−1^	Yes
	Jizan	66.3 ± 9.8%	− 0.45% decade^−1^	Yes
Surface wind speed (WS_10_, m s^−1^)	Tabuk	3.3 ± 2.2 m s^−1^	0.02 m s^−1^ decade^−1^	Yes
	Jeddah	3.7 ± 2.2 m s^−1^	0.02 m s^−1^ decade^−1^	Yes
	Jizan	3.3 ± 2.2 m s^−1^	0.01 m s^−1^ decade^−1^	Yes
Mean sea level pressure (SLP, hPa)	Tabuk	1009.9 ± 6.3 hPa	− 0.04 hPa decade^−1^	Yes
	Jeddah	1008.9 ± 4.5 hPa	− 0.11 hPa decade^−1^	Yes
	Jizan	1008.5 ± 4.3 hPa	− 0.08 hPa decade^−1^	Yes

The nonparametric Mann–Kendall test is used to detect monotonic trends in the corrected ERA5 data to examine whether the C_ERA5 data follow a significant (monotonic) trend.

The C_ERA5 data (1979–2020) confirmed a significant spatial long-term trend in T2m, RH and SLP. The long-term WS_10_ trend in Tabuk and Jeddah, however, was less notable than that in Jizan. The study area experiences a positive monotonic warming trend in the three studied cites. Similarly, WS_10_ attained a positive monotonic trend in the study area. In contrast, RH and SLP attained negative monotonic trends in the study area (Table 3).

In detail, the warming trend in Tabuk is more intense than that in Jizan, indicating that the T2 m difference across the study area may decrease in the future. Similarly, RH attains a much more significant negative trend in Jizan than that in Tabuk, indicating that the spatial RH variation in the study area may decrease in the future. In addition, the negative long-term SLP trend reaches its highest value in Jeddah, followed by Jizan and Tabuk, indicating that the SLP difference between Tabuk and Jeddah may decrease in the future, while the difference between Jeddah and Jizan may increase in the future.

##### Historical days

###### **Tabuk**

 The highest temperature of 44.87 °C in Tabuk was recorded on 12 July 2010 at 11:00 GMT, virtually on par with the value of 44.86 °C on 11 July 2010 at 13:00 GMT. However, the lowest temperature of − 2.93 °C in Tabuk was recorded on 15 January 2008 at 4:00 GMT, virtually on a par with the value of − 2.65 °C on 15 January 2008 at 5:00 GMT.

In Tabuk, the maximum RH value (= 93.9%) was recorded on 30 November 1989 at 02:00 GMT. In contrast, the minimum RH value (= 0.1%) in Tabuk was recorded on 24 April 1994 at 16:00 GMT.

In Tabuk, the wind speed peaked (= 22.66 m s^−1^) on 13 March 2020 at 11:00 GMT, virtually on a par with the value of 22.61 m s^−1^ on the same day at 10:00 GMT.

Tabuk’s highest SLP value reached 1030.56 hPa and was recorded on 5 January 1992 at 04:00 GMT, virtually on par with the value of 1030.56 hPa on the same day at 5:00 GMT. In contrast, Tabuk’s lowest SLP value reached 994.60 hPa and was recorded on 24 March 2019 at 11:00 GMT, virtually on par with the value of 994.64 hPa on the same day at 10:00 GMT.

###### Jeddah

 The highest temperature of 55.7 °C in Jeddah was recorded on 5 June 2013 at 9:00 GMT, while the lowest temperature of 10.2 °C in Jeddah was recorded on 13 January 1992 at 4:00 GMT and on 26 February 1992 at 02:00 GMT.

In Jeddah, the maximum RH value (= 86.2%) was recorded on 20 October 1980 at 02:00 GMT, on 7 October 1995 at 04:00 GMT, and on 16 September 1995 at 04:00 GMT.

In Jeddah, a wind speed peak (= 26.3 m s^−1^) was recorded on 28 March 1997 at 09:00 GMT, virtually on par with the value of 24.9 m s^−1^ on 18 December 1985 at 10:00 GMT.

Jeddah’s highest SLP value, 1024.9 hPa, was recorded on 26 February 1992 at 08:00 and 07:00 GMT. In contrast, Jeddah’s lowest SLP value reached 995.7 hPa and was recorded on 13 August 1998 at 0:00 GMT, virtually on par with the value of 995.8 hPa on 12 August 1998 at 0:00 GMT.

###### Jizan

In Jizan, the highest temperature of 47.2 °C was recorded on 11 July 1995 at 11:00 and 12:00 GMT, while the lowest temperature of 0.1 °C in Jizan was recorded on 7 February 1993 at 04:00 and 03:00 GMT.

In Jizan, the maximum RH value (= 100%) was recorded on 29 July 1995 at 10:00 GMT. However, a wind speed peak (= 16.6 m s^−1^) was recorded in Jizan on 17 July 2018 at 11:00 GMT.

Jizan’s highest SLP value of 1021.9 hPa was recorded on 5 January 1992 at 07:00 GMT virtually on par with the value of 1021.5 hPa on the same day at 06:00 GMT. However, Jizan’s lowest SLP value reached 997.0 hPa and was recorded on 29 July 2001 at 0:00 GMT, virtually on par with the value of 997.1 hPa on 22 June 2007 at 14:00 GMT.

##### Probability of occurrence

**Figure 10:**
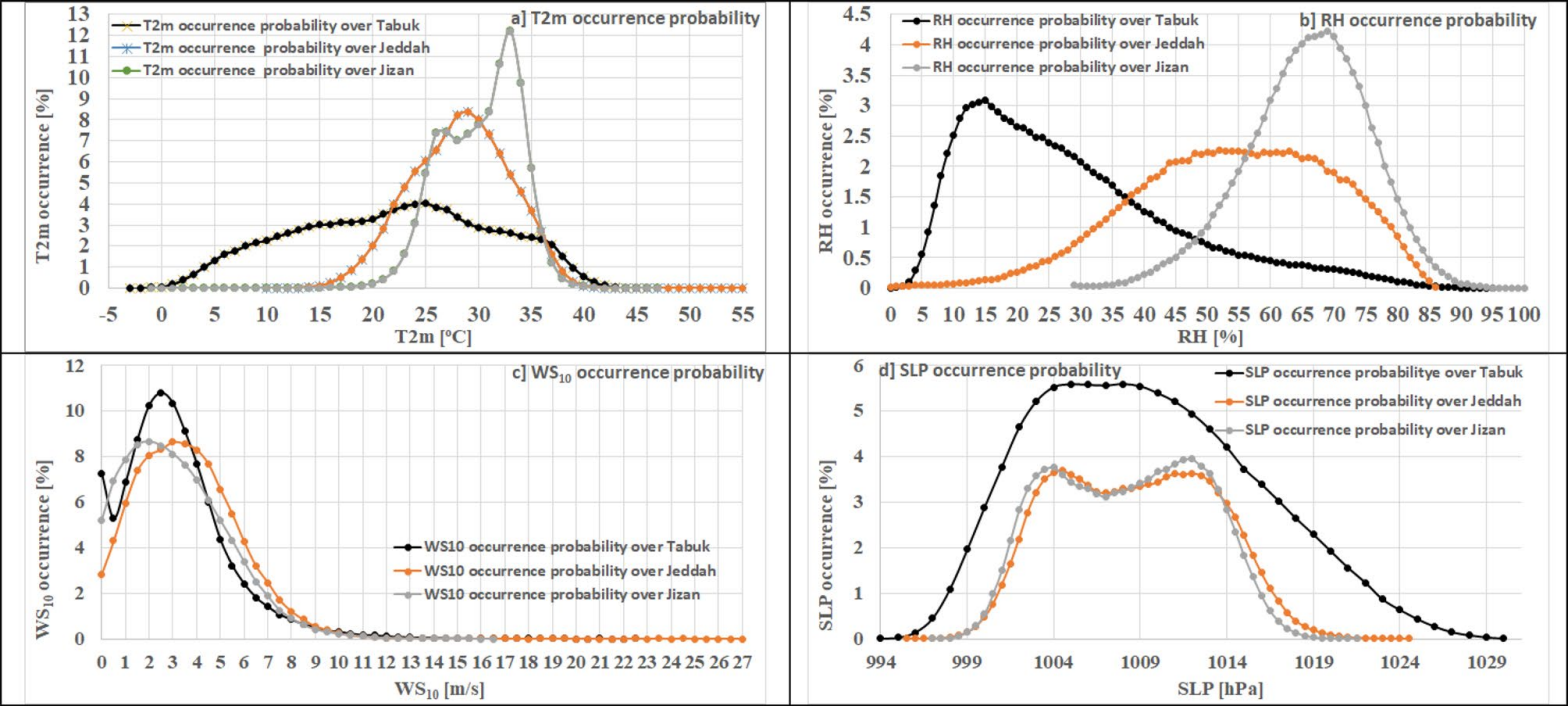
Occurrence probability of the parameters considered based on the hourly corrected ERA5 data (1979–2020) in the study area. Math Works, Inc. MATLAB. Version 2020a, Math Works, Inc., 2020. www.mathworks.com.

The highest values of the hourly T2m occurrence probability are 4.13% (24–25 °C), 8.4% (28–29 °C), and 12.2% (32–33 °C) in Tabuk, Jeddah and Jizan, respectively. In regard to the warmest hours (mean + 2*standard deviation), the occurrence probability is 0.46% (> 41.1 °C), 1.36% (> 37.8 °C), and 0.85% (> 38.1 °C) in Tabuk, Jeddah and Jizan, respectively. However, regarding the coldest hours (mean − 2*standard deviation), the occurrence probability is 2.3% (< 3.7 °C), 3.2% (< 19 °C), and 3.3% (< 23.3 °C) in Tabuk, Jeddah and Jizan, respectively, as shown in Fig. 10a.

The highest values of the hourly RH occurrence probability were 3.09% (15–16%), 2.26% (51–52%), and 4.22% (68–69%) in Tabuk, Jeddah and Jizan, respectively. Regarding the highest RH values (mean + 2*standard deviation), the occurrence probabilities were 5.7% (> 62.5%), 0.15% (> 85.5%), and 1.17% (> 85.9%) in Tabuk, Jeddah and Jizan, respectively. In addition, no hourly occurrences of the lowest RH value were noted (< 0%; mean − 2*standard deviation) in Tabuk. During approximately 3.1% (3.5%) of the time, the lowest hourly RH values < 23.5% (46%) were noted in Jeddah (Tabuk), as shown in Fig. 10b.

The hourly WS_10_ mostly (10.78%, 8.6%, and 8.6%) ranged from 2.5 to 3 m s^−1^, 3 to 3.5 m s^−1^, and 2 to 2.5 m s^−1^ in Tabuk, Jeddah and Jizan, respectively. In regard to the windiest WS_10_ hourly events (mean + 2*standard deviation), the occurrence probabilities were 4.7% (> 7.7 m s^−1^) in Tabuk, 3.6% (> 8.1 m s^−1^) in Jeddah, and 4.1% (> 7.7 m s^−1^) in Jizan. The calmest hourly events (< 0.5 m s^−1^) occurred approximately 12.5%, 9.9%, and 16.4% of the time in Tabuk, Jeddah and Jizan, respectively, as shown in Fig. 10c.

The hourly SLP mostly (6.7%, 3.7%, and 3.9%) ranged from 1012 to 1013 hPa, 1004.5 to 1005 hPa, and 1012 to 1012.5 hPa in Tabuk, Jeddah and Jizan, respectively. In regard to the highest SLP hourly events (mean + 2*standard deviation), the occurrence probabilities were 2.44% (> 1022.5 hPa), 1.23% (> 1017.9 hPa), and 0.86% (> 1017.1 hPa) in Tabuk, Jeddah and Jizan, respectively. The lowest hourly events (mean − 2*standard deviation) occurred during 0.6% (< 997 hPa), 0.3% (< 999 hPa), and 1% (< 999.9 hPa) of the time in Tabuk, Jeddah and Jizan, respectively, as shown in Fig. 10d.

##### Principal component analysis (PCA)

PCA (Table 4) revealed that only two principal components (PCs) accounted for 85%, 82% and 75% of the parameter variance in Tabuk, Jeddah and Jizan, respectively.

**Table 4:** Principal component analysis of Tabuk, Jeddah and Jizan based on the corrected ERA5 parameters.

Variables		Principal component (PC)			
		PC1	PC2	PC3	PC4
Tabuk	Surface air temperature (T2m)	− **0.59**	− 0.21	0.09	0.77
	Relative humidity (RH)	**0.52**	0.17	0.75	0.36
	Surface wind speed (WS_10_)	− 0.27	**0.96**	− 0.06	0.06
	Mean sea level pressure (SLP)	**0.55**	0.08	− 0.65	0.52
Jeddah	Surface air temperature (T2m)	− **0.69**	0.19	− 0.26	0.65
	Relative humidity (RH)	**0.44**	**0.63**	0.58	0.40
	Surface wind speed (WS_10_)	− 0.29	− **0.61**	0.72	0.16
	Mean sea level pressure (SLP)	**0.58**	− 0.44	− 0.29	0.62
Jizan	Surface air temperature (T2m)	− **0.69**	0.11	− 0.11	0.70
	Relative humidity (RH)	0.21	**0.70**	0.65	0.21
	Surface wind speed (WS_10_)	− 0.18	− **0.64**	0.74	0.04
	Mean sea level pressure (SLP)	**0.66**	− 0.29	− 0.12	0.68

The bold numbers indicate highly significant correlations above 0.40.

In detail, the first PC, which is responsible for 62.71% and 45% of the parameter variance in Tabuk and Jeddah, respectively, indicates a strong correlation with three of the examined variables. This first PC decreases with decreasing T2m but increases with increasing RH and SLP. This indicates that T2m, RH, and SLP are highly correlated in Tabuk and Jeddah. In Jizan, the first PC, which is responsible for 47% of the parameter variance, exhibits a strong correlation with only two of the examined variables. This first PC decreases with decreasing T2m but increases with increasing SLP.

The second PC, which is responsible for 22.11% of the parameter variance in Tabuk, can be considered a measure of WS_10_. In Jeddah and Jizan, the second PC is responsible for 39% and 28%, respectively, of the parameter variance. The second PC attains a strong correlation with only two of the examined variables, RH and WS_10_, indicating the strongest co-dependence between RH and WS_10_.

### Statistical downscaling for future projection

In this section, the results of the GFDL mini-ensemble mean simulations under the different RCP scenarios are investigated for Tas, RH, WS_10_, and SLP.

#### GFDL model bias correction over the control period, 2006—2020

The GFDL mini-ensemble mean underestimates (overestimates) Tas in Tabuk and Jizan (Jeddah), as indicated in Table 5. In addition, the GFDL mini-ensemble mean underestimates (overestimates) RH in Jeddah and Jizan (Tabuk). Moreover, the GFDL mini-ensemble mean underestimates (overestimates) WS_10_ in Tabuk and Jeddah (Jizan). In general, the GFDL mini-ensemble mean overestimates SLP in the three studied cities.

**Table 5:** Performance of the GFDL mini-ensemble mean during the control period (2006–2020) in Tabuk, Jeddah and Jizan: only the RCP2.6 scenario is considered.

	Annual GFDL mini-ensemble mean—annual corrected ERA5 data			
	Surface air temperature (T2m, °C)	Relative humidity (RH, %)	Surface wind speed (WS_10_, m s^−1^)	Mean sea level pressure (SLP, hPa)
Tabuk	− 4.7	**4.4**	− **0.76**	**7.6**
Jeddah	1	− 14	− 0.5	**2.1**
Jizan	− 3.7	− 15.8	**0.4**	**3.5**

The bold numbers (shaded numbers) indicate overestimation (underestimation).

**Figure 11:**
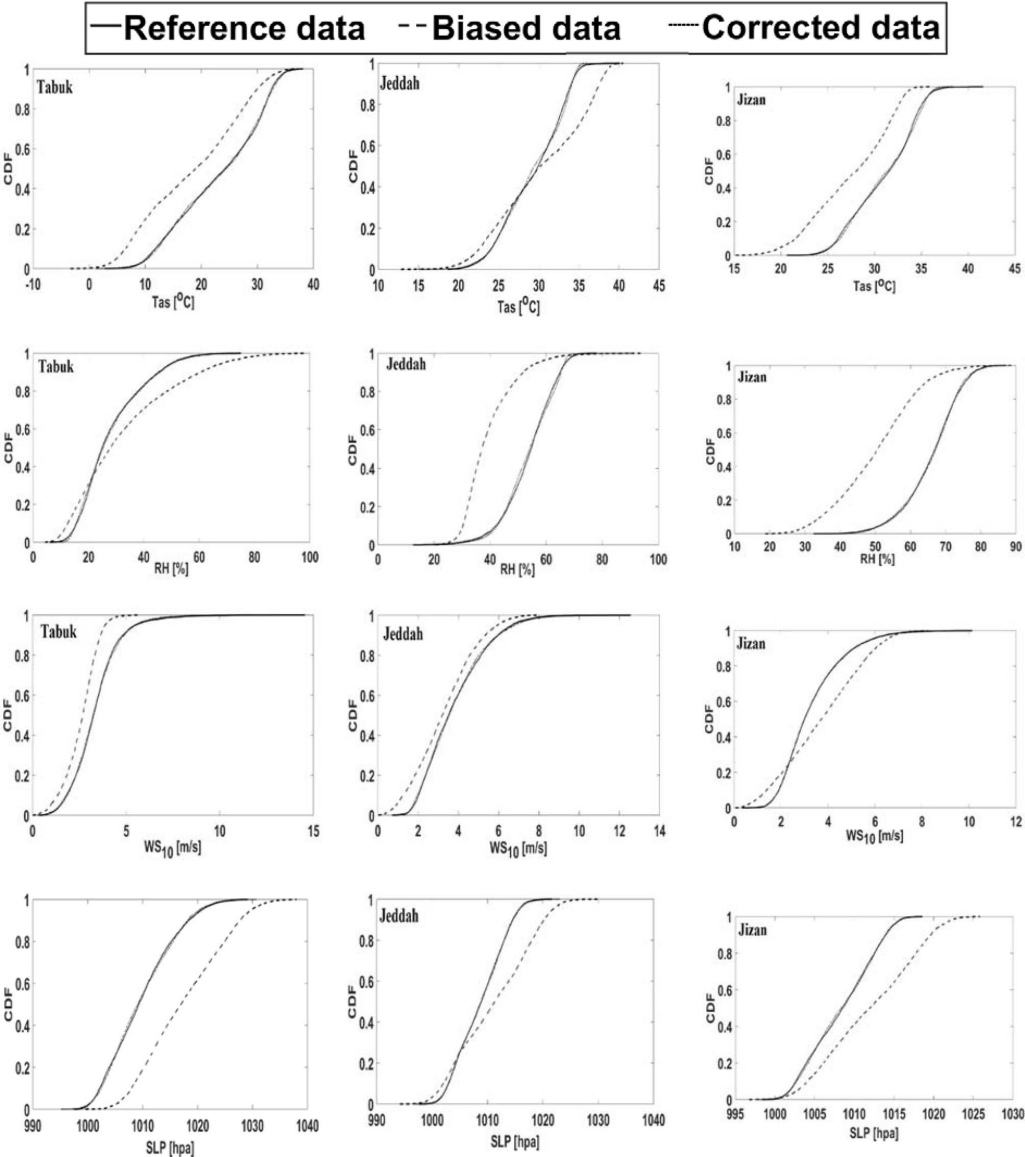
Cumulative distribution function (CDF) between the corrected ERA5 (C_ERA5) and GFDL mini-ensemble mean data over the control period (2006–2020) under the RCP2.6 scenario for the studied parameters. Tas (simulated 2-m air temperature), RH (relative humidity), surface wind speed (WS_10_), and SLP (mean sea level pressure). The corrected data curves are identical to the reference curves. Math Works, Inc. MATLAB. Version 2020a, Math Works, Inc., 2020. www.mathworks.com.

To overcome the above underestimation/overestimation of the GFDL mini-ensemble mean that is incomparable to the C_ERA5 data, a simple statistical model was developed by matching the CDF of the C_ERA5 data to that of the GFDL mini-ensemble mean data over the control period (2006—2020) under the different RCP scenarios, as shown in Fig. 11. Figure 11 shows the bias correction for only RCP2.6. However, the bias correction for RCP4.5, RCP6, and RCP8.5 is not shown due to the similarity with the RCP2.6 effect over the control period.

#### Future atmospheric parameters, 2006–2100

The GFDL mini-ensemble mean at each site over the 2006–2100 period was subjected to bias removal based on the developed statistical model, which was established by matching the CDF of the C_ERA5 data to that of the GFDL mini-ensemble mean data over the control period (2006–2020) under the different RCP scenarios. The resulting S_D_ GFDL mini-ensemble mean data were used to calculate the atmospheric future uncertainty with a high accuracy at each site (Tabuk, Jeddah and Jizan).

**Figure 12:**
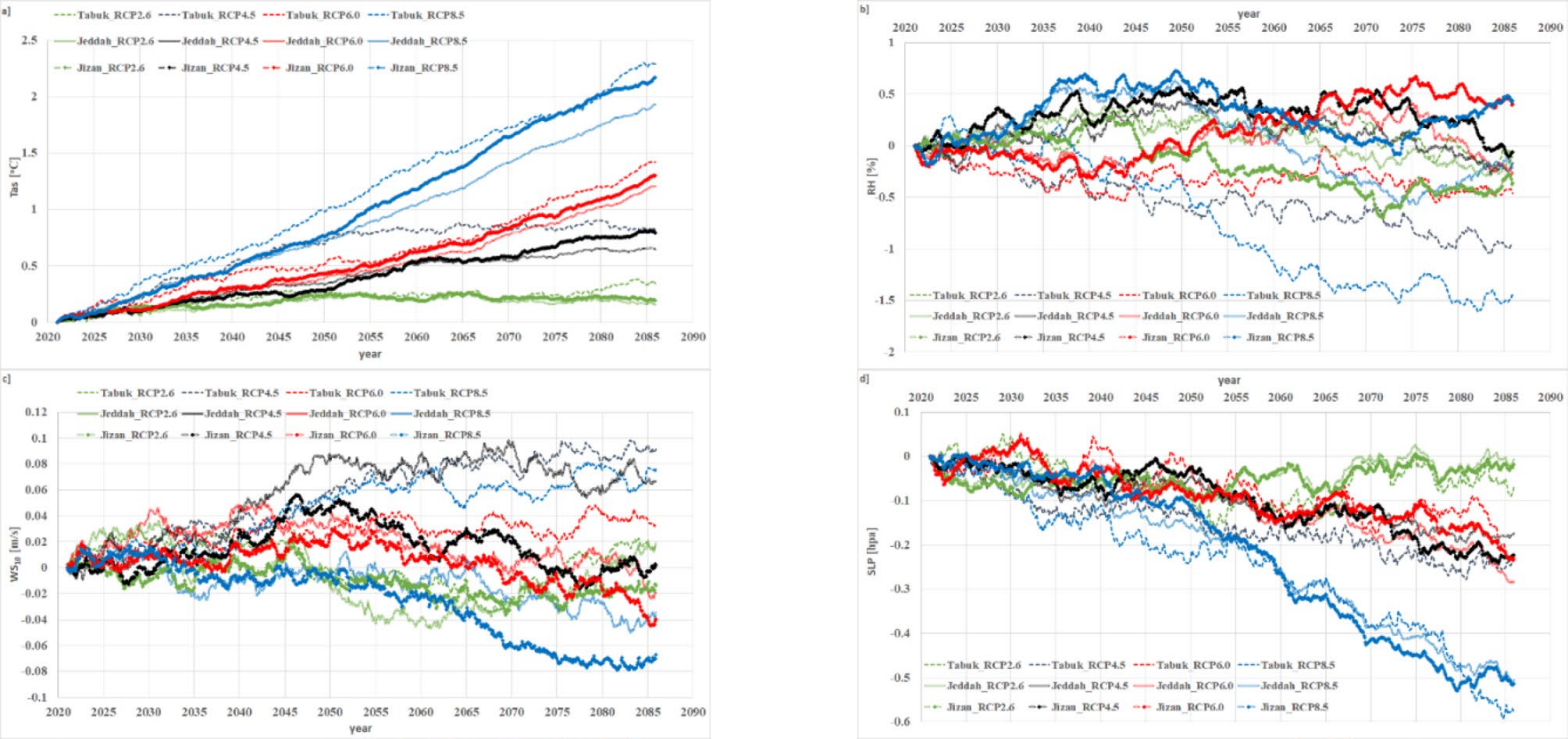
Thirty-year running annual means for (**a**) Tas (2-m air temperature), (**b**) RH (relative humidity), (**c**) WS_10_ (surface wind speed), and (**d**) SLP (mean sea level pressure) anomalies with reference to the 2006–2020 average values for statistical downscaling of the GFDL mini-ensemble mean simulations. Math Works, Inc. MATLAB. Version 2020a, Math Works, Inc., 2020. www.mathworks.com.

The S_D_ GFDL mini-ensemble means under the projected Tas scenarios in the current century indicate significant warming during the 2006–2100 period in the Saudi Arabia Red Sea coast, especially under the RCP8.5 scenario in Tabuk, as shown in Fig. 12a. The expected warming at the end of the current century (with reference to the 2006–2020 average value) ranges from 1.92 to 2.29 °C under the RCP8.5 scenario, 1.20–1.41 °C under the RCP6.0 scenario, 0.64–0.81 °C under the RCP4.5 scenario and 0.15–0.34 °C under the RCP2.6 scenario.

The S_D_ GFDL mini-ensemble means under the projected RH scenarios in the current century indicate a significant negative trend from 2006 to 2100 in the study area, especially under the RCP8.5 scenario in Tabuk, as shown in Fig. 12b. The expected decrease in RH up to 2100 (with reference to the 2006–2020 average value) ranges from 1.5 to 0.43% under the RCP8.5 scenario, 0.46–0.40% under the RCP6.0 scenario, 0.06–0.98% under the RCP4.5 scenario, and 0.11–0.36% under the RCP2.6 scenario.

The S_D_ GFDL mini-ensemble means under the projected WS_10_ scenarios in the current century indicate both negative and positive trends from 2006 to 2100 in the study area, especially under the RCP8.5 scenario in Jizan, as shown in Fig. 12c. The expected changes in WS_10_ up to 2100 (with reference to the 2006–2020 average value) averages − 0.14 m s^−1^ under the RCP8.5 scenario, − 0.07 m s^−1^ under the RCP6.0 scenario, − 0.08 m s^−1^ under the RCP4.5 scenario, and − 0.03 m s^−1^ under the RCP2.6 scenario.

The S_D_ GFDL mini-ensemble means under the projected SLP scenarios indicate a significant negative trend from 2006 to 2100 in the study area, especially under the RCP8.5 scenario in Tabuk, as shown in Fig. 12d. The expected changes in SLP up to 2100 (with reference to the 2006–2020 average value) reach 0.062 hPa under the RCP8.5 scenario, 0.051 hPa under the RCP6.0 scenario, 0.058 hPa under the RCP4.5 scenario, and 0.058 hPa under the RCP2.6 scenario.

Generally, the future warming uncertainty is 2.14 °C, where 86% (14%) of the uncertainty is associated with scenario design (regional variation). In regard to the future RH, the uncertainty is 2.3%, and 25% of the uncertainty is associated with scenario design, while 75% is associated with regional variation. In regard to the future WS_10_, the uncertainty is 0.23 m s^−1^, where 21% of the uncertainty is associated with scenario design, and 79% is associated with regional variation. Regarding the future SLP, the uncertainty is 0.56 hPa, and 93% of the uncertainty is associated with scenario design, while only 7% is associated with regional variation.

## Discussion and conclusions

As mentioned above, a limited number of scientific studies has been conducted on the climate in the study area. The current paper presented an overview to bridge the present gap in climate knowledge of the Saudi Arabian Red Sea coast, especially regarding future projections.

Based on hourly observed time series for T2m, RH, WS_10_ and SLP, the present research studied the short-term atmospheric changes in Tabuk, Jeddah and Jizan from 2016 to 2020. The observed time series was employed to remove the bias in ERA5 data and calculate C_ERRA5 data for the three studied cities from 1979 to 2020 via cumulative distribution functions (CDFs). The long-term C_ERA5 data were analysed statistically with the following techniques: trend analysis, historical days, probability of occurrence, and PCA. The C_ERRA5 data were employed to downscale GFDL mini-ensemble mean simulations and ensure relevance to Tabuk, Jeddah and Jizan with the CDF strategy. Finally, the S_D_ GFDL mini-ensemble mean was considered to analyse the uncertainty in the future Tabuk climate.

The results based on the observed data indicated that the air temperature increased meridionally from north (Tabuk) to south (Jizan), partly due to the amount of net absorbed solar energy. The warmest month was July in Tabuk and Jeddah, with average values of 33.3 °C and 33.95 °C, respectively. However, in Jizan, the hottest month occurred one month earlier (June), with an average value of 34.7 °C. The coldest month was January in Tabuk, Jeddah and Jizan, with average values of 11.3 °C, 23.87 °C and 26.9 °C, respectively. The lowest surface air temperature that was 12.5 °C (15.6 °C) lower than that in Jeddah (Jizan) indicated a much more intensive cooling process during cold months in Tabuk stemming from its mountainous nature. Similarly, the RH increased meridionally from Tabuk to Jizan. In contrast, there were no consistent patterns of meridional changes in the study area regarding WS_10_ and SLP. In addition, the prevailing annual wind direction was NW (12.2% of the time) in Tabuk, N (19.11% of the time) in Jeddah, and W (15.6% of the time) in Jizan. Moreover, the prevailing wind direction indicated significant monthly variability in the study area. Generally, the monthly T2m/RH variability was lower in Jeddah and Jizan than that in Tabuk, partially reflecting the importance of studying the dynamic mechanism that shapes the temperature variability near the Earth’s surface and upper layer.

Generally, the monthly T2m/SLP patterns were very close among the 3 studied cities. However, the monthly RH pattern in Tabuk was much closer to that in Jizan than to that in Jeddah. In the same manner, the monthly RH pattern in Tabuk was much closer to that in Jizan than to that in Jeddah.

The ERA5 data described the real atmospheric characteristics of the Saudi Arabian Red Sea Coast in a reasonable way, as CDF bias correction against real observations was insignificant in 66% of the study cases. This high performance indicates significant spatial variation, reaching a maximum performance in Jeddah and a lowest performance in Jizan. In detail, the highest match for the prevailing wind direction between the observed and ERA5 data was detected in Jeddah (Shift = 0), while in Tabuk, the shift was 22.5°, and finally, in Jizan, the shift was 45°. In contrast, ERA5 describes the present observation with a higher accuracy in Tabuk regarding T2m and Rh. However, a higher ERA5-based accuracy regarding WS_10_ and SLP was detected in Jeddah. This variability between the observed and ERA5 data initiates a future discussion regarding the reliability of ERA5 data versus land use and topography data in the study area.

In Tabuk, the present calculated long-term T2m annual mean (22.4 ± 9.3 °C) is approximately 1 °C higher than that modelled by^13^. This difference may be related to the different time spans. However, the current calculation of the long-term WS_10_ annual mean (3.3 ± 2.2 m s^−1^) is close to that modelled by^14^. In contrast, the C_ERA5 annual wind speed is approximately 50% lower than that previously calculated by^15^. This significant difference mainly occurs because^15^) the calculations were based on climate data, while the current calculations were based on hourly data.

The annual mean (from 1979 to 2020) of the C_ERA5 data in the study area confirms the occurrence of an 8.3 °C spatial T2m variation ranging from 22.4 to 30.7 °C, a 37.2% spatial RH variation ranging from 29.1 to 66.3%, a 0.4 m s^−1^ spatial WS_10_ variation ranging from 3.2 to 3.7 m s^−1^, and a 1.4 hPa spatial variation ranging from 1008.5 to 1009.9 hPa.

In addition, the C_ERA5 data revealed that T2m and WS_10_ attained a significant positive trend. However, RH and SLP exhibited a significant negative trend. The significance of the calculated trends was tested with the Mann–Kendall test. In detail, the Saudi Arabian Red Sea coast exhibits a significant spatial warming trend (from 1979 to 2020) for T2m (0.33–0.49 °C decade^−1^), RH (− 0.34 to − 0.45% decade^−1^), WS_10_ (0.01–0.02 m s^−1^ decade^−1^) and SLP (− 0.04 to − 0.11 hPa decade^−1^). However, the Saudi Arabian Red Sea Coast exhibits a significant spatial variation in the T2m trend, and RH and SLP may lead to a decrease in the spatial variation in the annual average values. The T2m and RH values in the south remain higher than those in the north, and the SLP value in the south remains lower than that in the north.

A historical warming day in the study area was recorded in Jeddah (55.7 °C) on 5 June 2013 at 9:00 GMT, while a historical cold day was recorded in Tabuk (− 2.93 °C) on 15 January 2008 at 4:00 GMT. In contrast, a peak RH value (100%) occurred in Jizan on 29 July 1995 at 10:00 GMT. However, an hourly wind speed peak (= 26.3 m s^−1^) was recorded in Jeddah on 28 March 1997 at 09:00 GMT. The highest SLP value reached 1030.56 hPa and was recorded in Tabuk on 5 January 1992 at 04:00 GMT, while the lowest SLP value reached 994.60 hPa and was recorded in Tabuk on 24 March 2019 at 11:00 GMT.

In general, the probability occurrence patterns indicate a significant spatial variation in each studied parameter. In addition, the C_ERA5 long-term records (1979–2020) confirm that T2m, RH and SLP in the study area are rarely below 3.7 °C, 1%, and 997 hPa, respectively, or above 41.1 °C, 85.9% and 1022.5 hPa, respectively. Moreover, WS_10_ rarely exceeds 8.1 m s^−1^.

At least 75% of the variance in the studied parameters can be explained by only two principal components in the study area, much more markedly in Tabuk (85%). In Tabuk and Jeddah, T2m, RH and SLP are closely co-dependent. However, in Jizan, only T2m and SLP are closely co-dependent.

The comparison between the GFDL mini-ensemble mean and corrected ERA5 data over the control period (2006–2020) confirms that the GFDL mini-ensemble mean in general underestimates Tas, RH and WS_10_ with average values of − 2.4 °C, − 8.4%, and − 0.2 m s^−1^, respectively. Moreover, the GFDL mini-ensemble mean generally overestimates SLP with an average value of 4.5 hPa. Thus, the GFDL mini-ensemble mean is applied to project the future climate in the study area after bias correction with the CDF technique.

The statistical downscaling results indicate that the study area will experience significant warming trends and significant negative RH/SLP trends. The future WS_10_ exhibits a wide uncertainty range from a positive to a negative trend. Moreover, the emission assumptions (according to the RCP used) combined with regional variation impose a significant effect on the uncertainty in all the studied parameters. The uncertainty related to the simulation performed is negligible, as the research considers only GFDL mini-ensemble means.

To support future management in Tabuk, statistical downscaling was applied to improve the GFDL mini-ensemble mean results, which exhibits a coarse resolution (2° × 2°), to ensure applicability in Tabuk. The S_D_ GFDL mini-ensemble mean simulations over the current century revealed a significant increase in Tas and WS_10_ with decreasing RH and SLP.

## Future work

The current research provides the first scientific analyses based on the current and future climate conditions in the Saudi Arabian Red Sea Coast. The main result of the current paper confirmed that the study area will face dramatic climatic changes, and the responses in terms of Tas, RH, WS_10_, and SLP are now better identified. This information can comprise a powerful database needed to improve the future vision of Tabuk.

In future studies, the authors will expand the current work to study new stations distributed throughout the KSA to obtain a full picture of the future uncertainties in the KSA. Moreover, the use of a regional climate model as a scientific tool for dynamical downscaling merits our attention in future work.

Finally, the most important conclusion of the current research addresses the project statistics of the studied climatic parameters up to 2100 through the different RCP scenarios. The projected climate uncertainties are likely to yield many negative socioeconomic impacts, especially in agriculture, water demand, and health. Thus, scientists in the fields of agriculture, medicine, and climate change must work with specialists in the field of water resources and decision-makers to improve climate policies in the KSA to identify innovative ways to turn the expected future climate challenges into social and economic opportunities.

## Methods

This paper obtained observation data to describe the short-term atmospheric variability in T2m, relative humidity (RH), and surface wind field components (eastward wind (U10), northward wind (V10) and SLP) in Tabuk, Jeddah and Jizan. Moreover, the long-term characteristics of the studied atmospheric parameters were analysed based on the ERA5 database after bias removal. Regarding the projection of future scenarios, statistical downscaling of the GFDL mini-ensemble mean was performed to study the future uncertainty in the study area.

### Material

#### Observation data

An automated weather observing system (AWOS) in Tabuk (760-m height), Jeddah (15-m height), and Jizan (6-m height) was employed to collect hourly T2m, surface dew point temperature (d2m), U10, V10 and SLP data. AWOS data are freely available via https://datasource.kapsarc.org/explore/dataset/saudi-hourlyweather-data/information/?disjunctive.station_name&disjunctive.wind_direction_quality&disjunctive.wind_type&disjunctive.wind_speed_quality&disjunctive.sky_ceiling_quality&disjunctive.visibility_distance_quality&disjunctive.visibility_variability_quality&disjunctive.air_temperature_quality&disjunctive.air_temperature_dew_point_quality&disjunctive.atmospheric_sea_level_pressure_quality (last accessed on 1 February 2021). These AWOSs were installed and maintained according to WMO regulations. The observations were calibrated according to a WMO standard height of 2 m above sea level for T2m/d2m and 10 m above sea level for U10 and V10. These data were used to generate a full image of the current short-term dynamics (from 2016 to 2020) of the studied atmospheric variables while validating the ERA5 database.

#### ERA5 database

Hourly data for T2m, d2m, U10, V10 and SLP from 1979 to 2020 were extracted from the Copernicus Climate Change Service (C3S) website (https://cds.climate.copernicus.eu/cdsapp#!/dataset/reanalysis-era5-single-levels?tab=form).

ERA5 data, which are distributed by the Copernicus Climate Change Service (C3S) and produced by the European Center for Medium Range Weather Forecasts (ECMWF), were intended to mitigate earlier issues (e.g., ERA Interim and ERA40 data) and improve the study of atmospheric parameters^18,19^ with a finer grid resolution of 0.25° × 0.25° and hourly data. These data, after a comparison to observations, were considered to analyse the long-term trends of the studied atmospheric variables. These data were further used for statistical downscaling.

#### Statistical downscaling from 2006 to 2100

GFDL climate model simulation results of the surface air temperature (Tas) over the 2006–2100 period was extracted from three model realizations: GFDL-ESM2M^20,21^, GFDL-CM3^22^, and GFDL-ESM2G^20,21^. These simulations are available on the GFDL website (ftp://nomads.gfdl.noaa.gov/CMIP5/output1/NOAA-GFDL) for the above four CMIP5 emission scenarios with a coarse grid resolution of 2° × 2°.

### Methods

Analysis of the recent and future variabilities in T2m, relative humidity (RH), 10-m height wind speed (WS_10_), wind direction (WD_10_), and SLP requires three steps. The first step is to analyse the current short-term atmospheric system from 2016 to 2020 using observation data. The second step concerns the ERA5 database (based on observations, remote sensing, and modelled information), where these data are employed after validation against observation data to analyse the current long-term climate system from 1979 to 2020. The third step is to conduct statistical downscaling to project the future climate from 2020 to 2100.

Generally, the relative humidity (RH) is calculated according to^23^ based on T2m and d2m, as expressed in Eq. (1).

1 $$ RH = \frac{{100*\exp \left( {\frac{{17.625*d2m\left( {^{ \circ } {\text{C}}} \right)}}{{243.04 + d2m\left( {^{ \circ } {\text{C}}} \right)}}} \right)}}{{\exp \left( {\frac{{17.625*T2m\left( {^{ \circ } {\text{C}}} \right)}}{{243.04 + T2m\left( {^{ \circ } {\text{C}}} \right)}}} \right)}} $$

#### Observation

Statistical analysis (observed as short-term means at hourly, daily, and monthly scales) of T2m, RH, WS_10_, and SLP was performed to investigate the temporal variation. In addition, the wind rose method is applied to similarly describe the temporal variation in WD_10_.

The SLP data pertaining to Tabuk, Jeddah and Jizan contained notable missing data fractions of 19.41%, 19.37% and 19.67%, respectively. Thus, the monthly mean was calculated only if the hourly data covered at least 33% of the month. Missing data for T2m, RH, and WS_10_ were very rare among the three stations.

Observed short-term hourly means for the parameters studied were considered to compute average values of T2m at hourly intervals throughout the observation period. Similarly, observed short-term monthly means were computed by averaging all the hourly data on a monthly basis during the observation period.

#### ERA5

Direct comparisons of the hourly observed data and ERA5 data were conducted to assess the efficiency of ERA5 data in Tabuk, Jeddah and Jizan. Moreover, f and t tests at a 95% confidence level were executed to examine whether the ERA5 and observation data exhibited similar means and variances (from the same population). Moreover, the ERA5 data were subjected to CDF bias correction between the ERA5 and observation data from 2016 to 2020.

The present strategy for bias correction is to match the CDF of the observations to that of the ERA5 data. Many researchers have applied this strategy. Anagnostou et al.^24^ implemented the CDF strategy to statistically adjust satellite microwaves for monthly rainfall estimates. Furthermore, Wood et al.^25^ used the CDF technique for long-range hydrologic forecasting. Reichle and Koster^26^ employed this strategy to match the CDF between satellite retrievals and soil moisture estimates.

Linear trend analysis of the ERA5 data after correction (hereafter, C_ERA5) from 1979 to 2020 was conducted to characterize the current long-term climate in the three studied cities. Moreover, the nonparametric Mann–Kendall test^27,28^ was used to detect monotonic trends in the C_ERA5 data to examine whether these data followed a significant (monotonic) trend. The Mann–Kendall test is a nonparametric test that is suitable for all distributions except data subject to serial correlation. Serial correlation denotes the relationship between observations of the same variable with different lag periods. If the serial correlation of a given variable is zero, each observation is independent. Conversely, if the serial correlation approaches one, the observations are serially correlated, and the Mann–Kendall test cannot be applied to detect monotonic trends^29^. Thus, the trend-free prewhitening approach was applied to eliminate serial correlation in the surface air temperature and wind speed time series^30^.

In addition, historical days containing peak events were analysed across all parameters considered, based on C_ERA5 hourly data, to determine the occurrence time of maximum and minimum values.

Moreover, the probability of occurrence of hourly T2m values for each degree Celsius (°C) covering the full T2m range was calculated. Similarly, the probability of occurrence of hourly RH values was also calculated for each 1% increment and each 0.5-m s^−1^ increment in the hourly wind speed. In addition, the probability of occurrence of hourly SLP values was calculated for each 1-hPa increment.

Finally, principal component analysis (PCA) was applied to the four atmospheric parameters considered (T2m, RH, WS_10_, and SLP) from 1979 to 2020 to produce a linear combination of the original values to initiate ordination of these parameters^31^. PCA is an unsupervised mathematical method to reduce the original variables into smaller-dimension new variables referred to as principal components (PCs) that still contain the original variables. Jolliffe^32^ described the PCA method in great detail. Generally, PCA aims to reduce a data matrix with a certain number of columns without much loss of information through (1) calculating the mean values of each column, (2) calculating the anomalies in each column, (3) calculating the covariance matrix of the obtained anomaly matrix, and (4) calculating lists of eigenvalues and eigenvectors of the covariance matrix. The eigenvectors represent the components/directions of the calculated reduced matrix, whereas the eigenvalues represent the magnitudes of the directions. The first PC contains the maximum possible information (data with the most variance), followed by the second PC containing the maximum remaining information, etc.

#### Statistical downscaling for future projection

The results of the three GFDL realizations were averaged to calculate the GFDL mini-ensemble mean from 2006 to 2100. Next, the cumulative distribution function (CDF) of the C_ERA5 data was matched with the GFDL mini-ensemble mean over the control period (2006–2020) to establish a simple statistical model for bias removal. This statistical model was applied to statistically downscale the studied atmospheric parameters over the long term (2006–2100) under the different future RCP scenarios. The statistically downscaled GFDL mini-ensemble mean simulation results (hereafter, S_D_ GFDL mini-ensemble mean) were used to calculate the future atmospheric uncertainty with a suitable accuracy and validity in Tabuk, Jeddah and Jizan. Future uncertainties in the studied parameters under the four RCP scenarios were calculated based on the 30-year running average.
